# Identification and mapping of major-effect flowering time loci *Autoflower1* and *Early1* in *Cannabis sativa* L.

**DOI:** 10.3389/fpls.2022.991680

**Published:** 2022-09-21

**Authors:** Jacob A. Toth, George M. Stack, Craig H. Carlson, Lawrence B. Smart

**Affiliations:** Horticulture Section, School of Integrative Plant Science, Cornell University, Cornell AgriTech, Geneva, NY, United States

**Keywords:** hemp (*Cannabis sativa* L.), bulk segregant analysis (BSA), flowering time, autoflower, early flowering, continuous light

## Abstract

Flowering time is an important trait for all major market classes of hemp (*Cannabis sativa*), affecting yields and quality of grain, fiber, and cannabinoids. *C. sativa* is usually considered a short-day plant, flowering once night length reaches a critical threshold. Variations in flowering time within and across cultivars in outdoor grown populations have been previously identified, likely corresponding to genetic differences in this critical night length. Further, some *C. sativa* are photoperiod insensitive, colloquially referred to as “autoflowering.” This trait has anecdotally been described as a simple recessive trait with major impacts on phenology and yield. In this work, the locus responsible for the “autoflower” trait (*Autoflower1)*, as well as a major-effect flowering time locus, *Early1*, were mapped using bulked segregant analysis. Breeder-friendly high-throughput molecular marker assays were subsequently developed for both loci. Also detailed are the flowering responses of diverse cultivars grown in continuous light and the result of crossing two photoperiod insensitive cultivars of differing pedigree.

## Introduction

Hemp (*Cannabis sativa*) is a multi-use crop that is widely considered a photoperiod sensitive, short-day plant. Flowering time is important for all major market classes, and uniform flowering dates within a cultivar are essential for ease of harvest. Fiber hemp benefits from a long growing season, as harvest usually occurs around the flowering date, and early flowering results in a shorter vegetative growth phase to accumulate biomass (Salentijn et al., 2019). Grain hemp must flower early enough such that grain can be harvested before frost if growing in temperate latitudes, but precocious flowering can lead to severe yield penalties due to a lack of time to accumulate biomass that provides photosynthate for grain filling. This is especially challenging for subtropical and tropical latitudes where most days of the year have nights longer than the critical night threshold (Zhang et al., 2021). For cannabinoid production, as with grain production, precocious flowering may result in reduced floral biomass yield, while plants that do not flower by the end of the season fail to accumulate high concentrations of cannabinoids (Stack et al., 2021). Additionally, cannabinoid profiles change throughout the maturation of the inflorescence, making initiation of flowering an important factor in timing regulatory compliance testing and harvest (Toth et al., 2021; Zhang et al., 2021).

Previous work has outlined differences in photoperiod threshold across different cultivars, in controlled environments as well as field conditions (Stack et al., 2021; Zhang et al., 2021). It has also been well established that some plants are photoperiod insensitive (day neutral), a trait proposed to have been introgressed from high-latitude populations, which have been classified by some as a putative species, *Cannabis ruderalis* (McPartland, 2018). Photoperiod insensitivity is sometimes referred to colloquially as “autoflower” (Gloss, 2015). This trait has been suggested to be inherited in a simple, recessive, Mendelian fashion, but there are limited data on this in the peer-reviewed literature (Green, 2005). A patent covering molecular markers and biotechnological manipulation of genes responsible for “autoflower” is held by Phylos Biosciences (Phylos Bioscience, International Patent WO 2021/097496 A2). In addition to commercial high-cannabinoid “autoflower” cultivars, several grain cultivars, such as ‘FINOLA,’ have been referred to as “autoflowering” in the literature (Van Bakel et al., 2011), but have a distinct phenotype compared to photoperiod-insensitive high-cannabinoid cultivars in that the height of mature ‘FINOLA’ depends greatly on latitude (being shorter at lower latitudes), while “autoflower” high-cannabinoid cultivars do not appear to exhibit this phenomenon (Callaway, 2002; Yang et al., 2020; Stack et al., 2021). However, it is not clear if the genetic mechanism for photoperiod insensitivity is the same in all cultivars (Zhang et al., 2021). Diagnostic molecular markers and complementation assays could help resolve this question.

It is a well-established phenomenon that there is significant population structure in *C. sativa*, associated at least in part with recent breeding history and geography (Carlson et al., 2021). While there is ongoing debate on the specifics of the nature of this population structure (hindered in part because of the ease of intercrossing between subgroups of *C. sativa*), there is strong support for at least two subpopulations, which have been described as subspecies (McPartland, 2018). The two subspecies that have been described differ in end use and likely origin, with *C. sativa* ssp. *sativa* grown for grain and fiber originally in northern European latitudes and *C. sativa* ssp. *indica* grown for cannabinoid production originally in Southeast Asia, including India. Various other subpopulations have been described, including a distinct clade of *C. sativa* with geographic origins in China (Carlson et al., 2021; Ren et al., 2021). Different taxonomic classifications have also been proposed, including the putative species *C. ruderalis*, which has been considered the source of the “autoflower” trait in all *C. sativa* populations (Green, 2005).

Genetic pathways for the induction of flowering are largely conserved across dicot plants, with major photosensory, thermosensory, and age-related pathways converging on major floral integrator genes (Mouradov et al., 2002; Jung and Müller, 2009). These floral integrator genes, including CO, FT, SOC1, and FLC, result in the expression of floral meristem identity genes such as AP1 and LFY (Mouradov et al., 2002). The expression of these floral meristem identity genes result in a switch from a vegetative phase to a reproductive phase. Upstream of the major floral integrator genes are a host of other well-established genes including PRR37, casein kinase I, AP2 group genes, and others, which have been associated with flowering time in various plants including rice (Hori et al., 2013), sorghum (Murphy et al., 2011), and pepper (Yuan et al., 2021).

There has been extensive research into genes involved in photoperiod response and earliness *per se* in a variety of crops, aided by extensive work in Arabidopsis (Blümel et al., 2015). However, there has been relatively little research in this area in *C. sativa*. A recent genome-wide association study (GWAS) in fiber hemp implicated genes in the major photosensory, thermosensory, and age-related pathways as well as a host of transcription factors in time to flower (Petit et al., 2020). While the genes implicated in this study serve as a potential starting point, more study on the genetics of flowering time control in *C. sativa* is required for predictive breeding efforts. The conserved nature of flowering induction may ease the discovery of relevant genes in extant germplasm.

Our group previously identified several populations marketed as F_1_ hybrids (‘Umpqua’ and ‘Deschutes’) that have individuals with two distinct flowering times, approximately a month apart (Stack et al., 2021). This segregation ratio would be expected if one parent was heterozygous for a major effect flowering time locus while the other parent was homozygous at that locus. If this were the case, such a significant locus would be well suited for development of high-throughput molecular markers such as PACE (PCR Allele Competitive Extension, 3CR). As differences in flowering are not obvious when the plant is in an early vegetative state, early screening with molecular markers for this trait or the autoflower trait could be very useful (Toth et al., 2018).

For essentially qualitative traits controlled by major effect flowering time loci as described here, bulk segregant analysis (BSA) has been successfully used to map genes and generate molecular markers and related assays (Song et al., 2017). Bulk segregant analysis is a technique that utilizes the sequencing of pooled DNA samples from individuals with the same phenotype in contrasting groups in a segregating population, and has been used effectively in a range of crops including *C. sativa* (Ban and Xu, 2020; Welling et al., 2020). Bulk segregant analysis usually involves short-read sequencing and subsequent alignment to a reference genome, but BSA involving long reads and reference-free techniques have been developed (Nordström et al., 2013; Segawa et al., 2021). The number of individuals in the pools must be sufficiently large to randomize the association of all regions of the genome except the region or regions associated with the trait of interest. Compared with other methods of mapping, this technique has the advantage of obtaining whole genome sequences of the region of interest, alleviating the issue of ascertainment bias present in other methods of sequencing mapping populations such as single nucleotide polymorphism (SNP) chips or genotyping-by-sequencing (GBS). It is also cheaper and results in higher read depth than individually sequencing genomes, but multiple sequencing efforts would be required for mapping more than one trait. Bulk segregant analysis can also be conducted using pre-defined molecular markers instead of direct sequencing, but the decreasing cost of sequencing has made these approaches less common (Zhang et al., 2009; Becker et al., 2011).

Once sequencing data are obtained from contrasting pools, a comparison of regions that differ in allelic frequency can be performed (Magwene et al., 2011). In the case of a simple recessive trait in an F_2_ population, one pool should be homozygous for a region containing the causative gene, while the other pool should have an alternative allelic frequency of ∼33% in that region. In the case of a major gene in a backcross, one pool should be homozygous in a region and the other pool should be heterozygous. The difference between allele frequencies can be represented in a number of ways, including comparing the number of significantly different SNPs in a region determined through Fisher’s Exact Test, a G-Test statistic, or through the delta-allele frequency method (also called the delta-SNP method) which involves determining the difference in allele frequency directly (Zhang and Panthee, 2020).

## Materials and methods

### Field and greenhouse trials of populations segregating for flowering time

A population segregating for photoperiod insensitivity was developed by first crossing a female autoflower plant from a feminized (all-female) seed lot numbered KG9202 (generously provided by Kayagene, Hollister, CA, United States) with a late flowering, photoperiod sensitive ‘Otto II’ plant (generously provided by Edgar Winters, WinterFox Farms, Klamath Falls, OR) determined to be male and cannabinoid chemotype III using molecular markers (Toth et al., 2020) to produce F_1_ family GVA-H-19-1148. These parental cultivar populations were previously trialed in the 2019 Cornell high-cannabinoid hemp field trial (Stack et al., 2021). One selected photoperiod-sensitive female F_1_ plant (GVA-H-19-1148-002) was multiplied by rooting stem cuttings, then one ramet was treated with silver thiosulfate to induce male flowers that pollinated multiple genetically identical female plants to generate F_2_ seed labeled GVA-H-20-1080 (Carlson et al., 2021). This technique results in an entirely female, or feminized, population (Lubell and Brand, 2018).

A second population segregating for photoperiod insensitivity was ‘TJ’s CBG’ (generously provided by Stem Holdings Agri, Eugene, OR), which was evaluated in the 2020 Cornell CBG hemp field trials and displayed a CBG-dominant chemotype (chemotype IV).

For initial assessment of photoperiod insensitivity in the segregating populations, seeds of each were sown in potting mix in 50-cell SureRoot trays on December 16, 2019 and grown in a greenhouse with a 16L:8D light schedule. Eighty-eight healthy plants in each population were transplanted to one-gallon pots on February 3, 2020. While flowering was evident on some plants at this point, rating for terminal flowering as previously defined (Stack et al., 2021) was completed on March 23, 2020.

The high-CBD cultivar ‘Umpqua’ (generously provided originally by Industrial Seed Innovations) was evaluated in the Cornell high-cannabinoid hemp field trials in 2019 and 2020 where flowering time was carefully assessed. Details about the 2019 trial are available in Stack et al. (2021). The 2020 trial was executed using similar protocols, but with a different seed lot of ‘Umpqua’ generously provided by Arcadia Biosciences (Davis, CA). An additional 100 plants taken equally from both seed lots were transplanted outdoors on July 22, 2021 in a trial to evaluate flowering time in Geneva, NY using similar protocols. The 2021 flowering time field trial also included 96 individuals from the KG9202 × ‘Otto II’ F_2_ population GVA-H-20-1080 and 26 plants of ‘Hempress’ (generously provided by Point3 Farma, Center, CO). Height and wet biomass was recorded for each plant in population GVA-H-20-1080 in this trial. Additional populations segregating for photoperiod insensitivity were identified in the 2020 Cornell CBG hemp field trial.^1^

To evaluate photoperiod insensitivity across diverse germplasm, 50 seeds of each population were sown in potting mix in 50-cell SureRoot trays on April 20, 2021 unless otherwise noted and grown in a greenhouse under continuous supplemental lighting from high pressure sodium lamps. Flowering was assessed weekly. Male flowering was considered to have started when the length of internodes at the apex of the plant shortened and male buds were clearly visible at the growing tip.

A complementation cross was completed between two photoperiod-insensitive plants: pollen parent ‘Picolo’ (generously provided by Hemp Genetics International, Saskatoon, SK), and a homozygous *Autoflower1/Autoflower1* seed parent from the ‘Le Crème’ cultivar population (generously provided by Ventura Seed Company, Camarillo, CA, segregating 1:3 for photoperiod insensitivity). The F_1_ plants from this cross were grown under 16L:8D with a 1 h night break in 50-cell SureRoot trays alongside known photoperiod sensitive and insensitive cultivars. Ten plants from each population were established on January 5, 2022. ‘Auto CBD’ and ‘Le Crème’ were feminized populations with no males. ‘RN16’ was a dioecious photoperiod-sensitive high CBD hemp cultivar (Stack et al., 2021).

### Bulk segregant analysis sequencing

DNA was extracted using a Qiagen DNeasy 96-well kit from young leaf tissue collected from plants in population GVA-H-20-1080 and dried on silica gel. Two pools were created by combining equal amounts of DNA from 28 flowering, photoperiod-insensitive plants and 25 non-flowering, photoperiod-sensitive plants. Illumina TruSeq libraries with an insert size of ∼500 bp were constructed for each pool by the Cornell Institute of Biotechnology then paired end 151 bp sequencing was performed on the Illumina NextSeq 2000 platform with ∼35× coverage.

DNA was extracted from dried, milled floral biomass samples of ‘Umpqua’ as previously described (Toth et al., 2020) for 15 early flowering and 15 late-flowering plants from the 2019 and 2020 trials and from 15 early flowering and 15 late-flowering samples from the 2021 flowering time trial. Illumina TruSeq libraries were constructed for each phenological pool and sequenced on the Illumina NextSeq 2000 platform, as described above.

Reads were aligned to the CBDRx-cs10 (GCF_900626175.2) genome assembly (Grassa et al., 2021) using Geneious Prime software (Biomatters, Inc., San Diego, CA, United States) using the Geneious mapper algorithm at the fastest speed with three iterations. Variants were also called in the Geneious Prime environment (coverage > 3, minimum variant frequency > 0.05). Variant calls were exported and modified using a custom Python script to be compatible with PyBSASeq (Zhang and Panthee, 2020). PyBSASeq was run using the “BulksOnly” protocol, assuming an F_2_ population structure for GVA-H-20-1080 and a backcross population structure for ‘Umpqua’ bulks. Significant SNPs and regions were calculated using Fisher’s Exact Test, a G-Test statistic, and the delta-allele frequency method.

### PCR allele competitive extension (PACE) genotyping assays

PCR allele competitive extension assays were designed manually in the Geneious Prime environment. PACE reactions were run according to the product manual (3CR Bioscience Ltd., Essex, United Kingdom). Polymorphic SNP in the *Autoflower1* region identified as perfectly associated with photoperiod phenotype in GVA-H-20-1080 pools were converted to PACE markers (Table 1 and Supplementary Table 1). These were assayed across multiple populations including the individual plants that formed the pool, a field grown population of GVA-H-20-1080, cultivars segregating for flowering time when grown under field conditions, and diverse photoperiod-sensitive and photoperiod insensitive cultivars. Unless otherwise noted, 8 plants from each cultivar or population were tested. For wide-germplasm testing, the primer sets AUTO-2 and EAR<jrn>LY-1</jrn> were used.

**TABLE 1:** PACE primers designed for the *Autoflower1* (AUTO) and *Early1* (EARLY) loci.

Primer	Group/location	Primer sequence
**AUTO-1**	18464905	
FAM	WT	GAAGGTGACCAAGTTCATGCTATCCAGGGTCTGGCTTTAAAAA
HEX	AUTO	GAAGGTCGGAGTCAACGGATTATCCAGGGTCTGGCTTTAAAAT
REV		CCATAAAATGATAAGTACACTCTAC
**AUTO-2**	19701425	
FAM	WT	GAAGGTGACCAAGTTCATGCTTTGGACTTCACCAAATGAGCCC
HEX	AUTO	GAAGGTCGGAGTCAACGGATTTTGGACTTCACCAAATGAGCCT
REV		CTTCTAACCCTTTGCATGAATG
**AUTO-3**	19731625	
FAM	AUTO	GAAGGTGACCAAGTTCATGCTCACAAGAATAATGCCCAAGAT
HEX	WT	GAAGGTCGGAGTCAACGGATTCACAAGAATAATGCCCAAGAC
REV		CCTAGGTTGACATAGCCACCA
**AUTO-4**	19991224	
FAM	AUTO	GAAGGTGACCAAGTTCATGCTTCTCACTTTCTGTCTTTTTCCCT
HEX	WT	GAAGGTCGGAGTCAACGGATTTCTCACTTTCTGTCTTTTTCCCC
REV		TCACAGTCTCAACAGGAGTGG
**AUTO-5**	21536161	
FAM	AUTO	GAAGGTGACCAAGTTCATGCTTTTTCATTTTCGGTGGGGTTTC
HEX	WT	GAAGGTCGGAGTCAACGGATTTTTTCATTTTCGGTGGGGTTTT
REV		GGTTGGATGTTTCAGCTGAAG
**EARLY-1**	41445929	
FAM	EARLY	GAAGGTGACCAAGTTCATGCTGGATACTAGCCACTAGAAAGGTTT
HEX	WT	GAAGGTCGGAGTCAACGGATTGGATACTAGCCACTAGAAAGGTTG
REV		CGAAGGAGATAAAGACTGTGAG
**EARLY-2**	46288769	
FAM	EARLY	GAAGGTGACCAAGTTCATGCTATGTGTGTGTGCCTGTAGAACC
HEX	WT	GAAGGTCGGAGTCAACGGATTATGTGTGTGTGCCTGTAGAACT
REV		GTCCTAACCTTCAGAAACTCCTAG

Additional PACE primers segregating in GVA-H-20-1080 associated with Autoflower1 are listed in Supplementary Table 1.

## Results

### *Autoflower1* photoperiod insensitivity is a recessive Mendelian trait

Two populations segregating for photoperiod insensitivity (GVA-H-20-1080 and ‘TJ’s CBG’) were planted under non-inductive, long day conditions (16L:8D dark). In the GVA-H-20-1080 population, 28/88 plants flowered (31.8%), and in the ‘TJ’s CBG’ population, 24/88 plants flowered (27.3%). These data are not significantly different from 25% of the plants flowering (χ*^2^ P* > 0.05), consistent with a recessive allele at a single locus we are designating *Autoflower1* that was homozygous in KG9202, heterozygous in the photoperiod sensitive F_1_ progeny of KG9202 × ‘Otto II,’ and segregating 1:2:1 in the GVA-H-20-1080 F_2_ and ‘TJ’s CBG’ populations. The parents of the GVA-H-20-1080 population were also grown under these conditions, with KG9202 flowering alongside the *Autoflower1/Autoflower1* homozygotes and ‘Otto II’ not being induced to flower.

### Mapping of the *Autoflower1* locus

**FIGURE 1:**
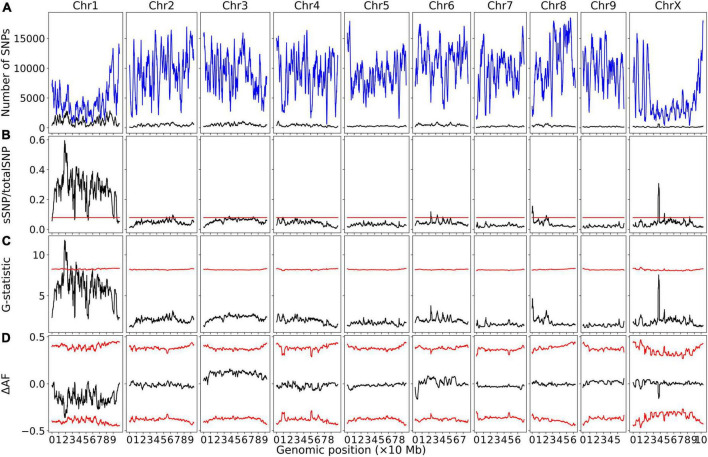
Bulk segregant analysis examining pools of photoperiod-insensitive and photoperiod-sensitive plants from GVA-H-20-1080. A physical genomic window size of 2 Mb and step size of 10 kb is shown. **(A)** Chromosomal distribution of SNPs. Total SNP number is in blue and significant SNP number (Fisher Exact Test *P* < 0.01) is in black. **(B)** Ratio of significant SNPs:Total SNPs across chromosomes. **(C)** G-Test statistic values across chromosomes. **(D)** Delta-allele frequency (ΔAF) values across chromosomes. Red lines represents significance thresholds determined by simulation of 10000 SNPs given allele depths at the 99.5th percentile for significant SNPs:Total SNPs and G-statistic, and the 99% confidence interval for ΔAF values.

Bulk segregant analysis of Illumina sequence pools of photoperiod-sensitive and -insensitive individuals showed clear statistical significance for the G-Test statistic in a region of Chromosome 1 (NC_044371.1) for population GVA-H-20-1080 (Figure 1). 230420 SNPs were included on Chromosome 1 in this analysis. No other chromosome reached significance by this metric (Figure 1). The significant region associated with *Autoflower1* spanned 17.74–22.94 Mb on Chromosome 1, with highly significant genomic windows (G-Test statistic > 11.5) at 18.59–19.70 Mb.

Using a delta-allele frequency approach, none of the genome reached significance, but the region identified on Chromosome 1 neared significance, and there were non-significant peaks on Chromosomes 8 and X.

### *Autoflower1* candidate gene analysis

Within the G-statistic significant region of *Autoflower1* on Chromosome 1 defined by the BSA of GVA-H-20-1080, 237 annotated genes were identified using the NCBI Genome Data Viewer. Of these, 75 were uncharacterized. Candidate genes potentially involved in controlling flowering time include: DOF zinc finger nucleases (LOC115704700, LOC115704742), nuclear transcription factor Y subunit B-1 (NFYB1, LOC115706176), floral homeotic protein APETALA 2 (AP2, LOC115708151), regulator of nonsense transcripts UPF2 (LOC115706264), zinc finger CCCH domain-containing protein 11 (LOC115706080), two-component response regulator-like PRR37 (PRR37, LOC115705128), protein FAR1-RELATED SEQUENCE 5-like (LOC115703878, LOC115703890), and protein LONG AFTER FAR RED 3 (LOC115705698). The 237 genes within the significant region are detailed in Supplementary Table 2.

### Germplasm screening with *Autoflower1* molecular assays

Within the region significantly associated with *Autoflower1*, some SNP alleles that were homozygous in the photoperiod-insensitive bulk and had an allele frequency of ∼33% in the photoperiod-sensitive bulk were converted to PACE assays and screened on diverse germplasm (Table 2). As photoperiod-sensitive plants may be heterozygous for *Autoflower1*, the *Autoflower1* marker assay was considered to be perfect if the homozygous allelic group associated with photoperiod-insensitive plants from the bulk was associated with photoperiod-insensitive plants only, while photoperiod-sensitive plants were either heterozygous or in the alternate homozygous allelic group.

**TABLE 2:** Genotype group calls for the *Autoflower1* locus by cultivar or population.

Cultivar or population	Source	*Autoflower1* ^1^	n	AUTO-1^2^ (18.464 Mb)	AUTO-2 (19.701 Mb)	AUTO-3 (19.731 Mb)	AUTO-4 (19.991 Mb)	AUTO-5 (21.536 Mb)
								
				CASPL4D1	NFYB1	AP2	PRR37	LOC11570 3889
‘Anka’	UniSeeds Inc.	WT/WT	4	A/A	C/C	C/C	T/T	T/T
Bish Feral	Bish Enterprises	Unknown	8	A/A	C/C	C/C	C/C	T/T
C16	Arcadia	WT/WT	2	A/A	C/C	C/C	T/T	T/T
‘CFX-2’	Hemp Genetics Intl.	Unknown	8	A/A	C/C	C/C	C/C	T/T
‘Henola’	Intl. Hemp	Unknown	8	A/A	C/C	C/C	T/T	T/T
‘Picolo’	Hemp Genetics Intl.	Unknown	16	A/A	C/C	C/C	Seg*	T/T
‘Puma’	CN Kenaf and Hemp	WT/WT	4	A/A	C/C	C/C	Seg*	T/T
RN13A	Paul Smith Denver Co.	WT/WT	4	A/A	C/C	C/C	T/T	T/T
RN17	Paul Smith Denver Co.	WT/WT	8	A/A	C/C	C/C	Seg*	T/T
‘Si-1’	CN Kenaf and Hemp	WT/WT	19	A/A	C/C	C/C	Seg*	T/T
‘Canda’	Parkland Ind.	Unknown	8	A/A	C/C	C/C	C/C	T/T
Missouri Feral	John Fike (40.2, −94.6)	Unknown	8	A/A	C/C	C/C	C/C	T/T
‘Nebraska’	Winter Fox Farms	Unknown	8	A/A	C/C	C/C	Seg*	T/T
‘NWG-Elite’	New West Genetics	Unknown	8	A/A	C/C	C/C	Seg*	T/T
‘T2’	Boring Hemp Co.	WT/WT	8	A/A	C/C	C/C	Seg*	T/T
‘USO-31’	UniSeeds Inc.	Unknown	8	A/A	C/C	C/C	C/C	T/T
‘CBG Delight’	Flura	Segregating	32	Seg†	Seg†	Seg†	Seg†	Seg*
‘H5’	American Hemp Co.	Segregating	32	Seg*	Seg†	Seg†	Seg*	Seg*
‘Hempress’	Point3 Farma	Segregating	24	Seg†	Seg†	Seg†	Seg*	Seg†
‘Le Crème’	Ventura Seed Co.	Segregating	44	ND	Seg†	Seg†	ND	ND
GVA-H-20-1080	Cornell Hemp	Segregating	184	Seg*	Seg†	Seg†	Seg†	Seg*
‘TJ’s CBG’	Stem Holdings Agri	Segregating	88	Seg†	Seg†	Seg†	Seg†	Seg*
‘Suver Haze’	Oregon CBD	WT/*Autoflower1*	8	T/C	T/C	T/C	C/C	C/T
‘Umpqua’	Ind. Seed Innovations	WT/ *Autoflower1*	4	T/C	T/C	T/C	T/T	T/C
AD1010	Phylos Bioscience	*Autoflower1*/ *Autoflower1*	4	T/T	T/T	T/T	T/T	Seg*
‘Alpha Explorer’	Phylos Bioscience	*Autoflower1*/ *Autoflower1*	4	T/T	T/T	T/T	T/T	Seg*’
‘Alpha Nebula’	Phylos Bioscience	*Autoflower1*/ *Autoflower1*	4	T/T	T/T	T/T	T/T	Seg*
‘Auto CBD’	Phylos Bioscience	*Autoflower1*/ *Autoflower1*	4	T/T	T/T	T/T	T/T	C/C
‘Auto CBG’	Oregon CBD	*Autoflower1*/ *Autoflower1*	4	T/T	T/T	T/T	T/T	Seg*
DNCBD	Arcadia Bioscience	*Autoflower1*/ *Autoflower1*	4	T/T	T/T	T/T	T/T	C/C
‘Dr. Chunk’	Kayagene	*Autoflower1*/ *Autoflower1*	4	T/T	T/T	T/T	T/T	C/C
‘Maverick’	Kayagene	*Autoflower1*/ *Autoflower1*	4	T/T	T/T	T/T	T/T	C/C
‘Purple Star’	Atlas Seeds	*Autoflower1*/ *Autoflower1*	4	T/T	T/T	T/T	T/T	C/C
‘Rincon’	Kayagene	*Autoflower1*/ *Autoflower1*	4	T/T	T/T	T/T	T/T	Seg*
‘Sour Citron’	Kayagene	*Autoflower1*/ *Autoflower1*	4	T/T	T/T	T/T	T/T	C/C
‘Sour RNA Seedless’ (triploid)	Oregon CBD	*Autoflower1*/ *Autoflower1/ Autoflower1*	4	T/T(/T)	T/T(/T)	T/T(/T)	T/T(/T)	C/C(/C)

^1^Expected Autoflower1 locus status based on phenotype and breeding history. Segregating populations all segregating 3:1 photoperiod sensitive: photoperiod insensitive.

^2^Seg†, segregating perfectly; Seg*, segregating imperfectly; ND, not determined.

### Effect of *Autoflower1* genotype on agronomic performance

**FIGURE 2:**
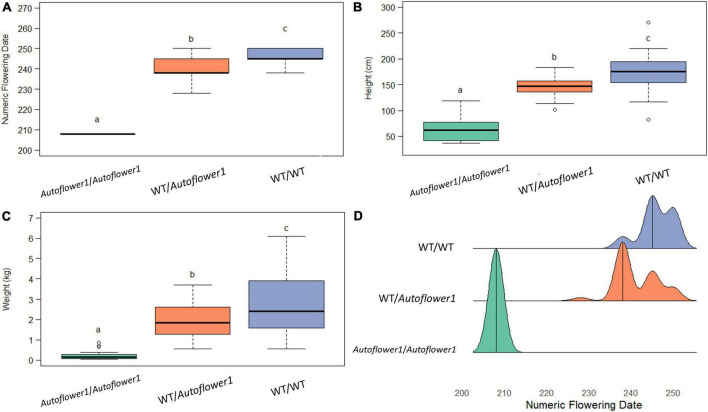
Effect of genotype at *Autoflower1* on agronomic traits. **(A)** Numeric (ordinal) flowering day. **(B)** Height, measured from base to tip at end of season. **(C)** Wet biomass. **(D)** Density ridge plot of flowering times within groups. Letters depict Tukey *post-hoc* test groupings (α = 0.05).

Ninety-six individuals of GVA-H-20-1080 grown in the 2021 flowering time field trial were genotyped at *Autoflower1* using the AUTO-2 marker to determine the additive effect of this locus when grown under field conditions. There was a significant effect of the allelic group on flowering date, height, and biomass, with heterozygotes being intermediate with respect to flowering date, height, and wet biomass (Figure 2).

### Flowering of diverse germplasm under continuous light

Several populations were grown under continuous light to determine if non-*Autoflower1/Autoflower1* cultivars could be induced to flower. There were distinct patterns of flowering time behavior among and within populations (Table 3). Most high-cannabinoid cultivars did not flower under continuous light, except for those homozygous for *Autoflower1*. Notably, plants heterozygous at the *Autoflower1* locus did not flower under continuous light. In general, cultivated fiber and Chinese cultivars did not terminally flower, while male and female plants in closely related feral populations did produce terminal and axillary (solitary) flowers, respectively. Also flowering under continuous light were the Canadian grain cultivars, ‘Picolo’ and ‘CFX-2’ (Hemp Genetics International, Saskatoon, SK).

**TABLE 3:** Time to flower of various *C. sativa* cultivars and populations under continuous light.

Cultivar or population	Males present^1^	Taxonomic group^2^	*AUTO -FLOWER1* ^1^	*EARLY1* ^1^	Source	Flowering date^1,3^				
						
						May 10	May 17	May 24	May 31	June 28
‘Carmagnola’	+	Fiber/Feral	–/–	–/–	Schiavi Seed	–	–	–	–	–
‘Puma’	+	Chinese	–/–	–/–	CN Kenaf and Hemp	–	–	–	–	–
GVA-H-19-1052	–	West Coast	–/–	+/+, ±, –/–	Cornell Hemp	–	–	–	–	–
RN16	+	T1/R4	–/–	–/–	Paul Smith Denver Co.	–	–	–	–	–
‘Umpqua’	–	West Coast	±	±, –/–	Arcadia Bioscience	–	–	–	–	–
NS52	–	Not tested	±, –/–	±, –/–	Phytonyx	–	–	–	–	–
‘Fedora 17’	–	Grain/Dual	–/–	+/+, ±, –/–	UniSeeds, Inc.	–	–	–	–	Axillary
‘A2R4’	+	Fiber/Feral	–/–	–/–	WinterFox Farms	–	–	–	–	Axillary
‘BaOx’	+	BaOx/Otto II	–/–	–/–	Ryes Creek	–	–	–	–	M, axillary F
‘Nebraska’	+	Fiber/Feral	–/–	–/–	WinterFox Farms	–	–	–	–	M, axillary F
Missouri Feral	+	Fiber/Feral	–/–	–/–	J. Fike	–	–	–	M only	M, axillary F
GVA-H-20-1080	–	Intercross	+/+, ±, –/–	–/–	Cornell Hemp	–	1/4	1/4	1/4	1/4
‘Auto CBD’	–	Not tested	+/+	–/–	Phylos Bioscience	–	+	+	+	+
‘Auto CBG’	–	Not tested	+/+	–/–	Oregon CBD	–	+	+	+	+
‘Socati Auto’	–	Not tested	+/+	–/–	Boring Hemp Co.	–	+	+	+	+
KG9202	–	West Coast	+/+	–/–	Kayagene	–	+	+	+	+
‘Anka’	+	Grain/Dual	–/–	–/–	UniSeeds Inc.	–	M only	M only	M, axillary F	M, axillary F
‘Henola’	–	Grain/Dual	–/–	+ /+	Bija Hemp	–	Some	+	+	+
‘CFX-2’	+	Grain/Dual	–/–	–/–	HGI	M only	+	+	+	+
‘Picolo’	+	Grain/Dual	–/–	–/–	HGI	M only	+	+	+	+

^1^XY plants present in population; yes (+), no (–); Alleles; non-WT (+), WT (–); Flowers present on date, yes (+), no (–).

^2^Taxonomical group data described in Carlson et al. (2021).

### Complementation test of photoperiod-insensitive cultivars

**FIGURE 3:**
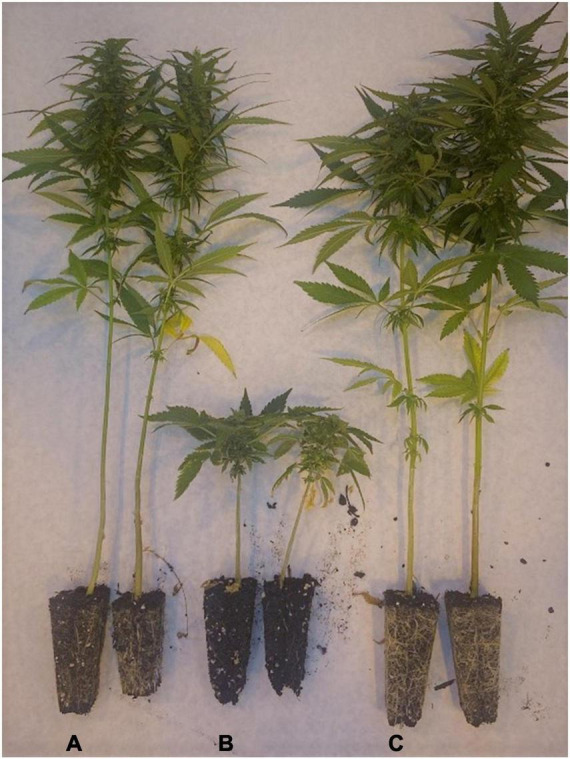
Photoperiod-insensitive *C. sativa* grown under long days (16L:8D) and photographed 85 days after planting. Representative female plants of panel **(A)** ‘Picolo,’ **(B)** ‘Le Crème’ (*Autoflower1*/*Autoflower1*), and **(C)** ‘Le Crème’ (*Autoflower1*/*Autoflower1*) × ‘Picolo’ F_1_. Receptive white pistils were present at the apex of plants in panels **(A,C)** at 85 days after planting, but only brown desiccated pistils were present on plants in panel **(B)**.

The Canadian grain hemp cultivar ‘Picolo’ and a subset of the individuals in the ‘Le Crème’ population both flowered under continuous light, but had contrasting PACE marker calls at the *Autoflower1* locus. A complementation test was performed to determine if there were distinct genes underlying their respective photoperiod insensitivity. All F_1_ plants from this cross were induced to flower, although the time to flower was distinct from the parents (Table 4). Homozygous *Autoflower1/Autoflower1* female plants flowered 3 weeks earlier than female ‘Picolo’ and female F_1_ plants, and male ‘Picolo’ plants flowered 2 weeks earlier than the male F_1_ plants. Female ‘Picolo’ and F_1_ plants were morphologically similar, while ‘Le Crème’ *Autoflower1* plants were distinct (Figure 3). All plants of a given sex and genotype flowered on the same day.

**TABLE 4:** Time to flower under long day (16L:8D) lighting.

Cultivar or pedigree	Males (weeks)	Females (weeks)
‘AutoCBD’, ‘Le Crème’ (*Autoflower1/Autoflower1)*	n/a	**4** (AutoCBD *n* = 10, Le Crème (*Autoflower1/ Autoflower1) n* = 3)
‘Picolo’	**4** (*n* = 7)	**7** (*n* = 3)
‘Le Crème’ (*Autoflower1/Autoflower1)* × ‘Picolo’ (F_1_)	**6** (*n* = 4)	**7** (*n* = 6)
RN16	None	None

### Segregation for flowering time in ‘Umpqua’

**FIGURE 4:**
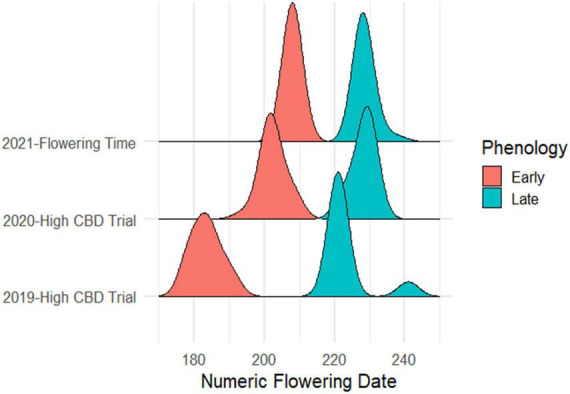
Density ridge plot of ‘Umpqua’ flowering time in the field over 3 years in Geneva, NY.

The cultivar ‘Umpqua’ has been grown in Cornell field trials in 2019, 2020, and 2021. In each year, two distinct flowering times were noted (Figure 4). Over the course of 3 years, clear grouping was apparent, with 78 plants total in the early flowering group and 97 plants total in the later flowering group. These data are consistent with a 1:1 segregation of early and late phenotypes (χ^2^ = 2.063, *P* = 0.15), characteristic of a backcross involving a major effect gene (designated here as *Early1*) that is heterozygous in one parent and homozygous in the other. Neither phenotype was induced to flower under continuous light (Table 3).

### Mapping of *Early1* in ‘Umpqua’

**FIGURE 5:**
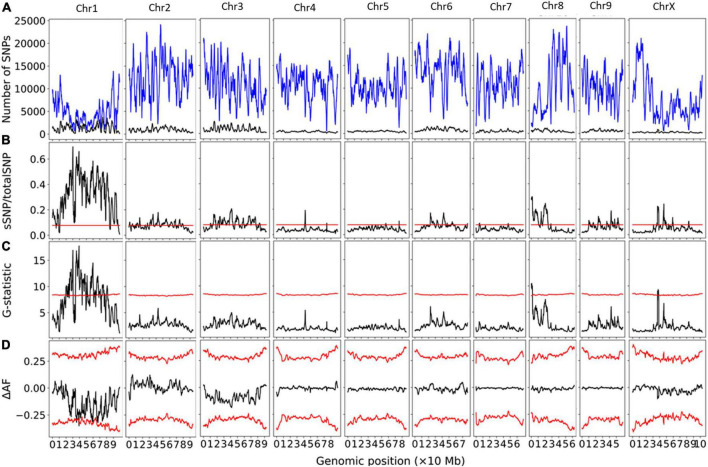
Bulk segregant analysis for pools of early- and late-flowering plants from ‘Umpqua.’ Physical genomic window size is 2 Mb and step size is 10 kb. **(A)** Chromosomal distribution of SNPs. Total SNP number is in blue and significant SNP number (Fisher Exact Test *P* < 0.01) is in black. **(B)** Ratio of significant SNPs:Total SNPs across chromosomes. **(C)** G-statistic values across chromosomes. **(D)** Delta-allele frequency (ΔAF) values across chromosomes. Red lines represents significance thresholds determined by simulation of 10000 SNPs given allele depths at the 99.5th percentile for significant SNPs:Total SNPs and G-statistic, and the 99% confidence interval for ΔAF values.

Bulk segregant analysis showed clear statistical significance for the *Early1* locus on Chromosome 1 (NC_044371.1), reaching significance using the delta-allele frequency approach (Figure 5). 247814 SNPs on Chromosome 1 were used in this analysis. Examination of the significant SNP data showed that the early flowering ‘Umpqua’ group was heterozygous at *Early1* while late flowering ‘Umpqua’ group was not.

Using a G-statistic threshold, most of Chromosome 1, as well as small peaks on Chromosomes 8 and X, were deemed significant.

### *Early1* candidate gene analysis

For the two ‘Umpqua’ pools, there were several small peaks that exceeded significance levels for the delta-allele frequency metric on Chromosome 1, spanning the intervals 35.26–36.23, 38.67–39.36, and 59.8–59.9 Mb. A total of 45 genes are annotated within these regions, of which the strongest candidate gene for *Early1* based on molecular function is LOC115705415 (annotated to encode Casein kinase 1-like protein 1), located on Chromosome 1 at 39.265 Mb (Supplementary Table 3). Polymorphic SNPs that were heterozygous for *Early1* in the early flowering pool were developed into high-throughput PACE marker assays (Table 1). Genotype assays correlated perfectly with the early- and late-flowering phenotypes of ‘Umpqua’ across all tested plants (*N* = 175).

## Discussion

### Autoflower1

*Autoflower1* confers photoperiod insensitivity in diverse *C. sativa* germplasm, and segregates in a simple, recessive (Mendelian) manner. Using BSA, we mapped the *Autoflower1* locus derived from KG9202 controlling the photoperiod insensitive phenotype in the GVA-H-20-1080 population to a small region on *C. sativa* Chromosome 1. Polymorphic markers from the Illumina data were used to develop *Autoflower1* molecular assays that accurately reported cultivars marketed as “Autoflowers,” and did not report any photoperiod sensitive plant as photoperiod insensitive. The markers were not effective at predicting photoperiod insensitivity across all germplasm, but this may be due to multiple causes of photoperiod insensitivity.

While *Autoflower1* is recessive with respect to photoperiod insensitivity, plants that were heterozygous for *Autoflower1* flowered approximately 2 weeks earlier than plants that were homozygous for *Autoflower1* under field conditions. This earlier flowering resulted in smaller plants with less total biomass, but may be useful for higher latitudes, as cultivars that are heterozygous for *Autoflower1* can produce very high yields in a shorter growing season (Stack et al., 2021). Many available cultivars are heterozygous for *Autoflower1*, which may be used as an effective breeding strategy for intellectual property protection. The prevalence of segregating populations marketed as cultivars suggests that some (perhaps unscrupulous or novice) breeders used parents that were heterozygous at *Autoflower1* leading to photoperiod-insensitive plants in the seed population. Populations segregating with ∼1/4 photoperiod-insensitive individuals, such as ‘TJ’s CBG,’ suggest production by a cross between two parents heterozygous for *Autoflower1*, possibly the self-pollination of a plant heterozygous for *Autoflower1*. As detailed in Table 2, several commercially marketed cultivar populations from multiple sources were segregating for *Autoflower1*, which may have resulted in poor cultivar performance for growers due to variation in photoperiod sensitivity.

Further work to identify the taxonomic origin of *Autoflower1* is pertinent. *Autoflower1* is often ascribed in the gray literature as derived from *C. ruderalis*, but the most recent and in-depth genomic studies do not support the existence of this group (Green, 2005; Carlson et al., 2021; Ren et al., 2021). *Autoflower1* would be expected to have evolved either at very high or very low latitudes, where photoperiod insensitivity is evolutionarily advantageous because seasonal variation in daylength is minimal. Further plant collecting expeditions and population genomics analyses would help resolve the evolutionary origin of *Autoflower1* as well as the extent of the genetic and phenotypic diversity of photoperiod sensitivity in *C. sativa*.

Future work to determine the causative gene at *Autoflower1* will allow biotechnological manipulation of the photoperiod sensitivity phenotype and more facile conversion of elite cultivars to and from photoperiod insensitivity. There were strong candidate genes for *Autoflower1* based on annotated predicted molecular function in the significant QTL interval identified for the GVA-H-20-1080 pools. Notably, SNPs near the genes for nuclear transcription factor Y subunit B-1 (NFYB1, LOC115706176) and floral homeotic gene APETALA 2 (AP2, LOC115708151) were in linkage disequilibrium and were perfectly associated with predicted trait phenotype across all individuals tested. These genes have the potential to be causative for the trait, as a Nuclear factor Y gene (DTH8) plays an important repressive role related to photoperiod in rice (Wei et al., 2010) while AP2 homologs are also important flowering time repressors in pepper (Yuan et al., 2021) and Arabidopsis (Yant et al., 2010). Future gene silencing or knockouts of these and other potential candidate genes may lead to identification of the true gene or set of genes responsible for this trait, although a patent is already held covering biotechnological manipulation of genes within this genetic interval (Phylos Bioscience, International Patent WO 2021/097496 A2).

### Continuous light

Plants from diverse germplasm had different flowering responses to continuous light. Photoperiod-sensitive high-cannabinoid cultivars and modern European and Chinese fiber cultivars did not flower under continuous light. Fiber cultivars have been selected for their ability to continue to grow vegetatively until late in the season, which maximizes stem biomass yield. Some feral populations, which are closely related to European fiber cultivars (Carlson et al., 2021), displayed male flowering, but not terminal female flowering, perhaps indicating some selective advantage to photoperiod-insensitive male flowering outside of cultivation. This may also reflect the ancestral genetics of the progenitors of these feral populations, but it is difficult to know the original provenance of their progenitors.

Despite not being reported by the *Autoflower1* markers, Canadian grain cultivars ‘Picolo’ and ‘CFX-2’ flowered readily under continuous light conditions. This could be due to the molecular markers not being polymorphic or effective in these populations, or due to an alternate genetic basis for photoperiod insensitivity. Different genetic mechanisms may be resolved with a complementation test. If the same gene was responsible for photoperiod sensitivity in ‘Picolo’ and *Autoflower1* ‘Le Crème,’ F_1_ progeny from an intercross should be uniformly photoperiod-insensitive. Otherwise, other genes, dominance, or epistasis may be involved. The results (Table 4) were inconclusive, as all plants flowered under long days, but the timing and architecture of flowering (Figure 3) suggests more complex genetic regulation in photoperiod-insensitive plants across broad germplasm.

### Early1

Beyond segregation for *Autoflower1*, several elite populations marketed as cultivars have been demonstrated as segregating 1:1 for a major-effect early flowering time phenotype (Stack et al., 2021). The *Early1* locus, which confers an apparent effect size of 2–4 weeks earlier flowering in ‘Umpqua,’ was also mapped to Chromosome 1 using BSA, but to a different location than *Autoflower1*. The apparent different effect in 2019 compared to 2020 and 2021 may have been due to differences in flowering time rating or non-uniform planting dates. Molecular markers for *Early1* were identified and high-throughput assays developed for this locus, which could further aid in development of cultivars with uniform flowering time.

In searching for candidate genes in the confidence interval for the *Early1* locus identified in ‘Umpqua’ populations, only a small portion of Chromosome 1 was found to exceed significance thresholds by the delta-allele frequency method. One possible candidate gene for early flowering within this small interval encodes a Casein kinase 1-like protein 1 (LOC115705415). This gene is homologous to the major flowering time gene *Early flowering 1*/*Heading date 16* in rice, another short day plant (Hori et al., 2013). Future validation work could involve genetic engineering or genome editing to accomplish gene knockout or gene knock-in to confirm loss or gain of function. The molecular markers and assays for *Early1* presented in this work will be considerably valuable in breeding. Studies to further explore the interactions between these two flowering time loci, *Autoflower1* and *Early1*, will likely lead to a better understanding of the genetics of flowering time and development of stable cultivars with unique flowering times. As early flowering ‘Umpqua’ plants were heterozygous for both traits, the progeny of an inbred population would be expected to form nine genotypic groups, whose phenotypes would reveal the role of epistasis between these loci.

There were some differences in the statistical outcomes of the BSA for *Autoflower1* in comparison to *Early1* in ‘Umpqua.’ The statistically significant region of *Early1* in ‘Umpqua’ as determined by the G-Test statistic was much larger and broader than that of *Autoflower1*. This is not surprising if this segregation truly is the result of a simple backcross, as recombination occurs only in one parent, rather than in both parents. This reduces the number of recombination events and therefore increases the apparent QTL size. However, analysis using the delta-allele frequency method resulted in a small peak and reliable diagnostic molecular assays were developed for the *Early1* locus.

Curiously, mapping both *Autoflower1* and *Early1* had apparent peaks on Chromosomes 8 and X, reaching statistical significance by the G-statistic threshold in the case of mapping *Early1*. It is unlikely that a 3-locus model explains the observed segregation in flowering time, so this is likely an experimental artifact. The unusual peaks may be due to errors in mapping or genome assembly, with segments on Chromosomes 8 and X being highly homologous to Chromosome 1 leading to inappropriate mapping, or errors in assembly with those significant regions actually residing on Chromosome 1. The CBDRx-cs10 genome used is known to be incomplete and may not be correctly physically ordered (Kovalchuk et al., 2020).

Many *C. sativa* cultivars produced during the rapid expansion of the cannabinoid industry were segregating for flowering time. There is a critical need in the industry to develop uniform and stable cultivars that represent a range of critical photoperiods. Cultivars with known critical photoperiods can be more effectively matched with the latitude of agricultural regions and environmental conditions in controlled environment cultivation. A better understanding of the genetic basis of flowering time in *C. sativa*, coupled with molecular tools to accelerate breeding and selection, will enable the development of new uniform cultivars to meet this need.

## Data availability statement

The data presented in this study are deposited in the NCBI SRA repository, accession numbers: SRR20046529-SRR20046532.

## Author contributions

JT: conceptualization, methodology, formal analysis, and writing—original draft. GS and CC: conceptualization, resources, and writing—review and editing. LS: conceptualization, supervision, writing—review and editing, and funding acquisition. All authors contributed to the article and approved the submitted version.

## Footnotes

1
https://hemp.cals.cornell.edu/resource/2020-cbg-hemp-cultivar-trial/
