# Supplementary material for: Identification and mapping of major-effect flowering time loci Autoflower1 and Early1 in Cannabis sativa L
Source: Front Plant Sci. 2022 Sep 21;13:991680. doi: 10.3389/fpls.2022.991680 (PMC9533707; doi:10.3389/fpls.2022.991680)
Supplement: Supplementary file 1 [file Data_Sheet_1.docx]

**Supplementary Material**

**Table S1.** Additional PACE assay primers found segregating in GVA-H-20-1080 for *Autoflower1*.

| **Primer Name** | **Group** | **Primer Sequence** |
| --- | --- | --- |
| **AUTO-6** |  |  |
| FAM | AUTO | GAAGGTGACCAAGTTCATGCTACAATTTTCAGGTTGAAGTTCACG |
| HEX | WT | GAAGGTCGGAGTCAACGGATTACAATTTTCAGGTTGAAGTTCACA |
| REV | 17,992,374 bp | CATGTTCAATAGACTGAAAACACAGG |
| **AUTO-7** |  |  |
| FAM | WT | GAAGGTGACCAAGTTCATGCTATTTACAGGATCATGTTCAGGATTC |
| HEX | AUTO | GAAGGTCGGAGTCAACGGATTATTTACAGGATCATGTTCAGGATTT |
| REV | 19,701,226 bp | TACTTTTGTGGATAATAAAGACAC |
| **AUTO-8** |  |  |
| FAM | WT | GAAGGTGACCAAGTTCATGCTAAATCGCACTAAATTTGCTTGG |
| HEX | AUTO | GAAGGTCGGAGTCAACGGATTAAATCGCACTAAATTTGCTTGA |
| REV | 19,730,918 bp | CTAAGTATGTCTCACACTCCTG |
| **AUTO-9** |  |  |
| FAM | WT | GAAGGTGACCAAGTTCATGCTTCTGTATAATGGTGCTGCTATAA |
| HEX | AUTO | GAAGGTCGGAGTCAACGGATTTCTGTATAATGGTGCTGCTATAT |
| REV | 19,812,889 bp | CAGATATACAATCAGCAGTTCAGG |
| **AUTO-10** |  |  |
| FAM | AUTO | GAAGGTGACCAAGTTCATGCTCTTTGGTCGACCCTTTTTTGGCC |
| HEX | WT | GAAGGTCGGAGTCAACGGATTCTTTGGTCGACCCTTTTTTGGCT |
| REV | 20,703,247 bp | CAGCTCGATGGGAGAGTTTTAAG |

**Table S2.** Annotated genes within the G-test significant C. sativa Chromosome 1 region of the Autoflower1 locus in KG9202.

| **Gene ID** | **Start** | **End** | **Gene Annotation** |
| --- | --- | --- | --- |
| LOC115705412 | 17757915 | 17761814 | 26S proteasome non-ATPase regulatory subunit 7 A |
| LOC115705413 | 17761898 | 17763259 | uncharacterized LOC115705413 |
| LOC115708410 | 17787969 | 17788075 | small nucleolar RNA R71 |
| LOC115704794 | 17809134 | 17811013 | aquaporin PIP1-2-like |
| TRNAK-CUU | 17810460 | 17810532 | N/A |
| LOC115703844 | 17860930 | 17861292 | uncharacterized LOC115703844 |
| LOC115705898 | 17861734 | 17870351 | uncharacterized LOC115705898 |
| TRNAD-GUC | 17904682 | 17904753 | N/A |
| LOC115706071 | 17919663 | 17921702 | general transcriptional corepressor CYC8-like |
| LOC115708269 | 17976187 | 17979044 | protein yippee-like |
| LOC115705688 | 17994036 | 18001546 | bifunctional purine biosynthesis protein PurH |
| LOC115703846 | 18004646 | 18014013 | uncharacterized LOC115703846 |
| LOC115706360 | 18013921 | 18018088 | proteoglycan 4 |
| LOC115706362 | 18018874 | 18021131 | clathrin light chain 2 |
| LOC115706923 | 18086527 | 18088357 | uncharacterized LOC115706923 |
| LOC115703847 | 18111469 | 18116010 | glucan endo-1,3-beta-glucosidase-like |
| LOC115707100 | 18133132 | 18135405 | uncharacterized LOC115707100 |
| LOC115703848 | 18138092 | 18138795 | uncharacterized LOC115703848 |
| TRNAK-CUU | 18174222 | 18174294 | N/A |
| LOC115708200 | 18174349 | 18177391 | dolichyl-phosphate beta-glucosyltransferase-like |
| LOC115703849 | 18186228 | 18187327 | epsin-3-like |
| LOC115705535 | 18187626 | 18190236 | actin |
| LOC115703850 | 18217309 | 18219776 | CASP-like protein 4D2 |
| LOC115703851 | 18281470 | 18283194 | uncharacterized LOC115703851 |
| LOC115703853 | 18291896 | 18292757 | CASP-like protein 4D1 |
| LOC115707613 | 18296987 | 18297774 | CASP-like protein 4D1 |
| LOC115704700 | 18319699 | 18321222 | dof zinc finger protein DOF3.6-like |
| LOC115707297 | 18381741 | 18387836 | uncharacterized LOC115707297 |
| LOC115704699 | 18393658 | 18394370 | uncharacterized LOC115704699 |
| LOC115707222 | 18397696 | 18399429 | uncharacterized LOC115707222 |
| LOC115703854 | 18400528 | 18402632 | adenosine deaminase-like protein |
| LOC115704742 | 18431424 | 18433582 | dof zinc finger protein DOF3.6 |
| LOC115707796 | 18455778 | 18456591 | CASP-like protein 4D1 |
| LOC115703855 | 18463090 | 18463934 | CASP-like protein 4D1 |
| LOC115703856 | 18467789 | 18469378 | CASP-like protein 4D2 |
| LOC115704910 | 18481235 | 18483648 | actin |
| LOC115704911 | 18483833 | 18485141 | epsin-3-like |
| LOC115703857 | 18486944 | 18488635 | uncharacterized LOC115703857 |
| LOC115707581 | 18490400 | 18493444 | dolichyl-phosphate beta-glucosyltransferase |
| TRNAK-CUU | 18493497 | 18493569 | N/A |
| LOC115706390 | 18529902 | 18532213 | uncharacterized LOC115706390 |
| LOC115705614 | 18550573 | 18555132 | glucan endo-1,3-beta-glucosidase |
| LOC115706272 | 18590256 | 18592335 | uncharacterized LOC115706272 |
| LOC115704992 | 18900705 | 18901809 | zinc finger protein ZAT12 |
| LOC115703858 | 18915701 | 18917143 | uncharacterized LOC115703858 |
| LOC115708554 | 18921169 | 18921275 | small nucleolar RNA R71 |
| LOC115705890 | 18940084 | 18942251 | WAT1-related protein At2g39510-like |
| LOC115705892 | 18974401 | 18976327 | WAT1-related protein At2g39510 |
| LOC115708453 | 19004801 | 19004904 | small nucleolar RNA R71 |
| LOC115705891 | 19033360 | 19035330 | WAT1-related protein At2g39510 |
| LOC115708167 | 19068823 | 19070752 | WAT1-related protein At2g39510-like |
| LOC115704023 | 19128938 | 19130917 | transcription factor bHLH114-like |
| LOC115704024 | 19131885 | 19133568 | actin-depolymerizing factor 2 |
| LOC115704021 | 19134177 | 19137480 | protein ALP1-like |
| LOC115707008 | 19143232 | 19144512 | protein UPSTREAM OF FLC |
| LOC115707046 | 19145357 | 19148341 | nucleolin 1 |
| LOC115704213 | 19173526 | 19176502 | receptor-like protein kinase THESEUS 1 |
| LOC115704214 | 19176467 | 19178091 | F-box protein At2g39490 |
| LOC115706369 | 19193319 | 19195723 | photosynthetic NDH subunit of lumenal location 1, chloroplastic |
| LOC115706368 | 19195927 | 19203939 | ABC transporter B family member 6 |
| LOC115703860 | 19214594 | 19216857 | uncharacterized LOC115703860 |
| LOC115707202 | 19217473 | 19223517 | transcription initiation factor TFIID subunit 6 |
| LOC115707264 | 19229654 | 19234420 | spliceosome-associated protein 130 A |
| LOC115706831 | 19250006 | 19253484 | caffeoylshikimate esterase |
| LOC115706075 | 19260772 | 19265413 | protein-tyrosine-phosphatase MKP1-like |
| LOC115706691 | 19269185 | 19276537 | beta-hexosaminidase 1-like |
| LOC115705550 | 19279579 | 19291479 | protein GRIP |
| LOC115705052 | 19302758 | 19304050 | probable membrane-associated kinase regulator 4 |
| LOC115703861 | 19314102 | 19320767 | ankyrin repeat-containing protein ITN1-like |
| LOC115707983 | 19342709 | 19347249 | protein-tyrosine-phosphatase MKP1 |
| LOC115707988 | 19347725 | 19349376 | uncharacterized LOC115707988 |
| LOC115707986 | 19354466 | 19362100 | beta-hexosaminidase 1 |
| LOC115707984 | 19368217 | 19380104 | probable DNA double-strand break repair Rad50 ATPase |
| LOC115707987 | 19381034 | 19403194 | probable membrane-associated kinase regulator 4 |
| LOC115707985 | 19411191 | 19415240 | ankyrin repeat-containing protein ITN1 |
| LOC115708435 | 19447336 | 19447440 | small nucleolar RNA R71 |
| LOC115708463 | 19453408 | 19453514 | small nucleolar RNA R71 |
| LOC115706635 | 19526596 | 19531022 | uncharacterized LOC115706635 |
| LOC115706681 | 19586800 | 19591181 | uncharacterized LOC115706681 |
| LOC115708399 | 19597631 | 19597737 | small nucleolar RNA R71 |
| LOC115708189 | 19623001 | 19626945 | protein NRT1/ PTR FAMILY 2.7 |
| LOC115703863 | 19670607 | 19672347 | uncharacterized LOC115703863 |
| LOC115706683 | 19675794 | 19679721 | protein NRT1/ PTR FAMILY 2.7-like |
| LOC115706176 | 19691506 | 19696923 | nuclear transcription factor Y subunit B-1 |
| LOC115704691 | 19712612 | 19715469 | probable RNA-binding protein ARP1 |
| LOC115708151 | 19726723 | 19728921 | floral homeotic protein APETALA 2 |
| LOC115703865 | 19778639 | 19780198 | uncharacterized LOC115703865 |
| LOC115703866 | 19782063 | 19783840 | uncharacterized LOC115703866 |
| LOC115706264 | 19802609 | 19815150 | regulator of nonsense transcripts UPF2 |
| LOC115703868 | 19822088 | 19823007 | uncharacterized LOC115703868 |
| LOC115703869 | 19826131 | 19827204 | uncharacterized LOC115703869 |
| LOC115706080 | 19843513 | 19847204 | zinc finger CCCH domain-containing protein 11 |
| LOC115703870 | 19849983 | 19850489 | uncharacterized LOC115703870 |
| LOC115703871 | 19860264 | 19863668 | protein TONNEAU 1a-like |
| LOC115705128 | 19985933 | 19992033 | two-component response regulator-like PRR37 |
| LOC115705129 | 19988482 | 19992665 | uncharacterized LOC115705129 |
| LOC115704703 | 20010950 | 20018438 | TBC1 domain family member 8B |
| LOC115705441 | 20032520 | 20036951 | CDP-diacylglycerol--glycerol-3-phosphate 3-phosphatidyltransferase 2 |
| LOC115705487 | 20574051 | 20576803 | uncharacterized LOC115705487 |
| LOC115704827 | 20582024 | 20583639 | uncharacterized LOC115704827 |
| LOC115706793 | 20588649 | 20591700 | uncharacterized LOC115706793 |
| LOC115703873 | 20595436 | 20599191 | uncharacterized LOC115703873 |
| LOC115708318 | 20601551 | 20602941 | uncharacterized LOC115708318 |
| LOC115708319 | 20603094 | 20604601 | uncharacterized LOC115708319 |
| LOC115704823 | 20609630 | 20610957 | uncharacterized LOC115704823 |
| LOC115708152 | 20612149 | 20612975 | uncharacterized LOC115708152 |
| LOC115708215 | 20615998 | 20619859 | WD repeat-containing protein WRAP73 |
| LOC115706210 | 20624018 | 20631775 | nucleolar complex protein 2 homolog |
| LOC115706652 | 20640845 | 20644771 | protein IQ-DOMAIN 1-like |
| LOC115705663 | 20653407 | 20659939 | calcium-binding mitochondrial carrier protein SCaMC-1-like |
| LOC115707338 | 20664332 | 20664739 | low temperature-induced protein lt101.2 |
| LOC115704698 | 20667500 | 20669307 | LOB domain-containing protein 1 |
| LOC115708282 | 20696892 | 20698904 | uncharacterized LOC115708282 |
| LOC115705207 | 20713556 | 20727975 | Golgi to ER traffic protein 4 homolog |
| LOC115705208 | 20732258 | 20733326 | uncharacterized LOC115705208 |
| LOC115703874 | 20734426 | 20735177 | uncharacterized LOC115703874 |
| LOC115703875 | 20735420 | 20738200 | uncharacterized LOC115703875 |
| LOC115707794 | 20741920 | 20742444 | uncharacterized LOC115707794 |
| LOC115703876 | 20760091 | 20762582 | uncharacterized LOC115707794 |
| LOC115703877 | 20775753 | 20778199 | uncharacterized LOC115703877 |
| LOC115706729 | 20778199 | 20781809 | uncharacterized LOC115706729 |
| LOC115706745 | 20790932 | 20795500 | uncharacterized LOC115706745 |
| LOC115708543 | 20801164 | 20801270 | small nucleolar RNA R71 |
| LOC115703878 | 20816258 | 20818673 | protein FAR1-RELATED SEQUENCE 5-like |
| LOC115706783 | 20824032 | 20824510 | uncharacterized LOC115706783 |
| LOC115703879 | 20830310 | 20833207 | uncharacterized LOC115703879 |
| LOC115706770 | 20833217 | 20836949 | uncharacterized LOC115706770 |
| LOC115706785 | 20848052 | 20848331 | uncharacterized LOC115706785 |
| LOC115706767 | 20852425 | 20858895 | pre-rRNA-processing protein TSR1 homolog |
| LOC115706769 | 20861533 | 20868270 | phosphoglucomutase |
| LOC115706752 | 20868998 | 20871337 | uncharacterized LOC115706752 |
| LOC115706728 | 20874609 | 20881142 | endoplasmic reticulum metallopeptidase 1-like |
| LOC115706773 | 20885271 | 20889037 | mRNA-decapping enzyme subunit 2-like |
| LOC115706762 | 20892287 | 20897961 | DNA polymerase epsilon subunit 3-like |
| LOC115703880 | 20898688 | 20900527 | uncharacterized LOC115703880 |
| LOC115706743 | 20901023 | 20905614 | 3-hydroxyisobutyryl-CoA hydrolase-like protein 2, mitochondrial |
| LOC115706755 | 20944539 | 20947901 | aquaporin PIP2-2-like |
| LOC115703881 | 20957532 | 20960672 | bifunctional dihydrofolate reductase-thymidylate synthase 1-like |
| LOC115706734 | 20962955 | 20970736 | diaminopimelate decarboxylase 2, chloroplastic |
| LOC115706768 | 20977107 | 20983589 | pre-rRNA-processing protein TSR1 homolog |
| LOC115706771 | 20986209 | 20992938 | phosphomannomutase/phosphoglucomutase-like |
| LOC115703882 | 20996324 | 20998378 | uncharacterized LOC115703882 |
| LOC115706761 | 20998925 | 20999638 | protein PXR1-like |
| LOC115706753 | 21001989 | 21004329 | uncharacterized LOC115706753 |
| LOC115706766 | 21007607 | 21014141 | endoplasmic reticulum metallopeptidase 1-like |
| LOC115706748 | 21021481 | 21025532 | mRNA-decapping enzyme subunit 2 |
| LOC115706763 | 21030259 | 21033631 | DNA polymerase epsilon subunit 3 |
| LOC115706744 | 21044054 | 21048463 | 3-hydroxyisobutyryl-CoA hydrolase-like protein 2, mitochondrial |
| LOC115706754 | 21082797 | 21086224 | aquaporin PIP2-2 |
| LOC115706733 | 21099518 | 21104416 | bifunctional dihydrofolate reductase-thymidylate synthase |
| LOC115706735 | 21105580 | 21109352 | diaminopimelate decarboxylase 2, chloroplastic-like |
| LOC115703883 | 21134331 | 21139980 | phosphatidylinositol/phosphatidylcholine transfer protein SFH3-like |
| LOC115706760 | 21142406 | 21146635 | trafficking protein particle complex subunit 1 |
| LOC115703884 | 21147123 | 21147770 | uncharacterized LOC115703884 |
| LOC115706764 | 21152489 | 21155502 | uncharacterized LOC115706764 |
| LOC115706749 | 21155973 | 21157289 | caffeoylshikimate esterase |
| LOC115706727 | 21157426 | 21161133 | WPP domain-associated protein |
| LOC115706732 | 21165867 | 21168970 | asparagine--tRNA ligase, cytoplasmic 1 |
| LOC115703886 | 21171737 | 21172419 | sulfated surface glycoprotein 185 |
| LOC115706736 | 21178192 | 21184371 | patatin-like protein 6 |
| LOC115706741 | 21198455 | 21204613 | chorismate synthase, chloroplastic |
| LOC115703887 | 21270041 | 21271053 | uncharacterized LOC115703887 |
| LOC115706740 | 21328132 | 21332291 | protein IQ-DOMAIN 1 |
| LOC115706730 | 21354815 | 21362577 | nucleolar complex protein 2 homolog |
| LOC115706772 | 21371455 | 21375371 | WD repeat-containing protein WRAP73-like |
| LOC115706777 | 21378084 | 21381142 | uncharacterized LOC115706777 |
| LOC115703888 | 21381497 | 21382484 | uncharacterized LOC115703888 |
| LOC115706778 | 21386169 | 21387515 | uncharacterized LOC115706778 |
| LOC115706774 | 21401203 | 21402937 | uncharacterized LOC115706774 |
| LOC115706779 | 21409533 | 21410611 | uncharacterized LOC115706779 |
| LOC115706784 | 21410796 | 21412220 | uncharacterized LOC115706784 |
| LOC115706747 | 21416708 | 21419512 | uncharacterized LOC115706747 |
| LOC115706751 | 21433547 | 21437041 | 18S rRNA (guanine-N(7))-methyltransferase RID2 |
| LOC115706756 | 21437550 | 21440586 | general transcription factor IIF subunit 2 |
| LOC115706737 | 21447348 | 21462402 | beta-taxilin |
| LOC115706739 | 21474635 | 21477538 | elongation factor 1-alpha |
| LOC115706758 | 21477812 | 21479214 | uncharacterized LOC115706758 |
| TRNAP-AGG | 21479260 | 21479331 |  |
| LOC115706731 | 21483096 | 21486104 | heat shock protein 83 |
| LOC115706746 | 21487600 | 21512554 | RNA pseudouridine synthase 5 |
| LOC115706726 | 21512850 | 21522694 | uncharacterized LOC115706726 |
| LOC115703889 | 21535217 | 21536302 | uncharacterized LOC115703889 |
| LOC115706742 | 21542941 | 21546746 | mediator of RNA polymerase II transcription subunit 4 |
| LOC115708485 | 21548540 | 21548654 | U5 spliceosomal RNA |
| LOC115705698 | 21548989 | 21562933 | protein LONG AFTER FAR-RED 3 |
| LOC115705816 | 21576898 | 21580894 | serine carboxypeptidase-like 27 |
| LOC115708138 | 21587198 | 21589633 | tetraspanin-6 |
| LOC115705883 | 21631321 | 21636641 | MLO-like protein 12 |
| LOC115707955 | 21641087 | 21642766 | probable ubiquitin-conjugating enzyme E2 C |
| LOC115706419 | 21646660 | 21649551 | rhomboid-like protein 19 |
| LOC115705459 | 21651759 | 21659430 | uncharacterized protein slr1919 |
| LOC115704817 | 21678269 | 21680923 | serine/threonine-protein kinase-like protein CCR2 |
| LOC115705568 | 21707280 | 21711213 | UDP-glucuronic acid decarboxylase 6 |
| LOC115705371 | 21744345 | 21748626 | UDP-glucuronic acid decarboxylase 6 |
| LOC115706295 | 21762341 | 21764108 | 60S ribosomal protein L35 |
| LOC115705010 | 21783471 | 21786230 | psbP-like protein 1, chloroplastic |
| LOC115705011 | 21786352 | 21788085 | 60S ribosomal protein L23a |
| LOC115705469 | 21798933 | 21806810 | sulfate transporter 4.1, chloroplastic |
| LOC115708356 | 21844601 | 21844707 | small nucleolar RNA R71 |
| LOC115707642 | 21862431 | 21868753 | methionine--tRNA ligase, chloroplastic/mitochondrial |
| LOC115707643 | 21868959 | 21873269 | UDP-glucuronic acid decarboxylase 6 |
| LOC115707900 | 21922249 | 21922869 | uncharacterized LOC115707900 |
| LOC115708437 | 22181332 | 22181435 | small nucleolar RNA R71 |
| LOC115703890 | 22253112 | 22253711 | protein FAR1-RELATED SEQUENCE 5-like |
| LOC115703891 | 22256688 | 22258022 | putative nuclease HARBI1 |
| LOC115703892 | 22258693 | 22260784 | L10-interacting MYB domain-containing protein-like |
| LOC115705846 | 22262273 | 22267661 | cell division control protein 48 homolog C-like |
| LOC115706223 | 22293072 | 22298256 | cell division control protein 48 homolog C-like |
| LOC115703893 | 22301422 | 22301994 | uncharacterized mitochondrial protein AtMg00810-like |
| LOC115703894 | 22368938 | 22369380 | uncharacterized LOC115703894 |
| LOC115703895 | 22370786 | 22372795 | uncharacterized LOC115703895 |
| LOC115704323 | 22375502 | 22376137 | uncharacterized LOC115704323 |
| LOC115705857 | 22384916 | 22388552 | ATP-dependent Clp protease proteolytic subunit 3, chloroplastic |
| LOC115703896 | 22398199 | 22399764 | uncharacterized LOC115703896 |
| LOC115705147 | 22449283 | 22450610 | cysteine proteinase inhibitor 12 |
| LOC115705148 | 22450959 | 22455201 | dr1-associated corepressor-like |
| LOC115708407 | 22461826 | 22461932 | small nucleolar RNA R71 |
| LOC115705221 | 22464826 | 22470153 | KHG/KDPG aldolase |
| LOC115705220 | 22470184 | 22471754 | protein NDH-DEPENDENT CYCLIC ELECTRON FLOW 5 |
| LOC115705219 | 22471722 | 22474796 | protein SINE1 |
| LOC115707349 | 22533037 | 22537761 | dr1-associated corepressor |
| LOC115707350 | 22537889 | 22539186 | cysteine proteinase inhibitor 12 |
| LOC115703897 | 22563827 | 22567881 | uncharacterized LOC115703897 |
| LOC115703899 | 22596604 | 22599884 | ATP-dependent Clp protease proteolytic subunit 3, chloroplastic-like |
| LOC115708244 | 22613465 | 22615178 | allene oxide synthase 3 |
| LOC115706159 | 22629036 | 22632528 | WAT1-related protein At5g07050 |
| LOC115703900 | 22672126 | 22673222 | uncharacterized LOC115703900 |
| LOC115705549 | 22674151 | 22693965 | importin-11 |
| LOC115708272 | 22715424 | 22720089 | uncharacterized LOC115708272 |
| LOC115705717 | 22743955 | 22750802 | 3-ketoacyl-CoA synthase 19 |
| LOC115708458 | 22783056 | 22783160 | small nucleolar RNA R71 |
| LOC115708379 | 22789195 | 22789301 | small nucleolar RNA R71 |
| LOC115704792 | 22836060 | 22837352 | putative mitochondrial carrier protein PET8 |
| LOC115707033 | 22841731 | 22842155 | low temperature-induced protein lt101.2 |
| LOC115703903 | 22844971 | 22845690 | LOB domain-containing protein 1-like |
| LOC115703753 | 22848599 | 22849205 | plant UBX domain-containing protein 10-like |
| LOC115708325 | 22870648 | 22872672 | uncharacterized LOC115708325 |

**Table S3.** Annotated genes within the delta-allele frequency significant C. sativa Chromosome 1 region of the Early1 locus in ‘Umpqua’.

| **Gene ID** | **Start** | **End** | **Gene Annotation** |
| --- | --- | --- | --- |
| LOC115705813 | 38708527 | 38713025 | uncharacterized LOC115705813 |
| LOC115707485 | 38724607 | 38726891 | syntaxin-22 |
| LOC115706081 | 38729916 | 38733199 | transcription factor IIIA |
| LOC115705986 | 38735468 | 38738063 | histone acetyltransferase MCC1 |
| LOC115704029 | 38740394 | 38741329 | protein EXORDIUM-like 6 |
| LOC115706076 | 38742998 | 38744151 | THO complex subunit 7B-like |
| LOC115706077 | 38745459 | 38746158 | S-norcoclaurine synthase 2 |
| LOC115706078 | 38745617 | 38748388 | uncharacterized LOC115706078 |
| LOC115705063 | 38755581 | 38756697 | S-norcoclaurine synthase 2 |
| LOC115704031 | 38781233 | 38781825 | S-norcoclaurine synthase 2-like |
| LOC115704032 | 38785491 | 38786161 | S-norcoclaurine synthase 2-like |
| LOC115703764 | 38794465 | 38796045 | uncharacterized LOC115703764 |
| LOC115705164 | 38801599 | 38802357 | uncharacterized LOC115705164 |
| LOC115707475 | 38803330 | 38804310 | S-norcoclaurine synthase 2 |
| LOC115706447 | 38804614 | 38806086 | S-norcoclaurine synthase 2 |
| LOC115704033 | 38936030 | 38936745 | uncharacterized LOC115704033 |
| LOC115704231 | 39032675 | 39034746 | protein DETOXIFICATION 51 |
| LOC115707502 | 39059472 | 39060592 | uncharacterized LOC115707502 |
| LOC115705112 | 39238848 | 39240715 | uncharacterized LOC115705112 |
| LOC115705415 | 39265477 | 39269512 | casein kinase 1-like protein 1 |
| LOC115704841 | 39272873 | 39273746 | uncharacterized LOC115704841 |
| LOC115704840 | 39274362 | 39279949 | diacylglycerol O-acyltransferase 1B |
| LOC115705447 | 39292464 | 39316101 | eukaryotic initiation factor 4A-10 |
| LOC115705449 | 39309335 | 39315575 | THO complex subunit 6-like |
| LOC115705450 | 39353098 | 39353685 | uncharacterized LOC115705450 |
| LOC115704035 | 39353865 | 39355789 | THO complex subunit 6-like |
| LOC115706124 | 35279033 | 35283655 | beta-galactosidase 1-like |
| LOC115706126 | 35284120 | 35287188 | uncharacterized LOC115706126 |
| LOC115706125 | 35287250 | 35292827 | beta-galactosidase |
| LOC115706137 | 35289677 | 35290530 | uncharacterized LOC115706137 |
| LOC115706129 | 35421311 | 35422996 | GATA transcription factor 21-like |
| LOC115706127 | 35944099 | 35946237 | myb family transcription factor APL-like |
| LOC115706135 | 35950263 | 35952789 | uncharacterized LOC115706135 |
| LOC115704001 | 35977958 | 35978491 | uncharacterized LOC115704001 |
| LOC115705480 | 36069682 | 36070734 | wound-induced protein 1 |
| LOC115703563 | 36147501 | 36150228 | uncharacterized LOC115703563 |
| LOC115705850 | 36151362 | 36155217 | alpha-soluble NSF attachment protein 2 |
| LOC115708134 | 36160041 | 36163153 | amino acid transporter AVT6C |
| LOC115707593 | 36196345 | 36198771 | amino acid transporter AVT6C |
| LOC115704180 | 59843988 | 59844515 | uncharacterized LOC115704180 |
| LOC115706631 | 59864864 | 59867947 | eukaryotic translation initiation factor 2 subunit alpha homolog |
| LOC115704181 | 59868903 | 59871189 | probable methyltransferase PMT23 |
| LOC115704182 | 59871724 | 59872621 | 26S proteasome non-ATPase regulatory subunit 10-like |
| LOC115704768 | 59896681 | 59899567 | auxin-responsive protein IAA27 |
